# Lentiviral vectors encoding tetracycline-dependent repressors and transactivators for reversible knockdown of gene expression: a comparative study

**DOI:** 10.1186/1472-6750-7-41

**Published:** 2007-07-16

**Authors:** Krzysztof Pluta, William Diehl, Xian-Yang Zhang, Robert Kutner, Agnieszka Bialkowska, Jakob Reiser

**Affiliations:** 1 Gene Therapy Program, Department of Medicine, Louisiana State University Health Sciences Center, New Orleans, LA 70112, USA

## Abstract

**Background:**

RNA interference (RNAi)-mediated by the expression of short hairpin RNAs (shRNAs) has emerged as a powerful experimental tool for reverse genetic studies in mammalian cells. A number of recent reports have described approaches allowing regulated production of shRNAs based on modified RNA polymerase II (Pol II) or RNA polymerase III (Pol III) promoters, controlled by drug-responsive transactivators or repressors such as tetracycline (Tet)-dependent transactivators and repressors. However, the usefulness of these approaches is often times limited, caused by inefficient delivery and/or expression of shRNA-encoding sequences in target cells and/or poor design of shRNAs sequences. With a view toward optimizing Tet-regulated shRNA expression in mammalian cells, we compared the capacity of a variety of hybrid Pol III promoters to express short shRNAs in target cells following lentivirus-mediated delivery of shRNA-encoding cassettes.

**Results:**

RNAi-mediated knockdown of gene expression in target cells, controlled by a modified Tet-repressor (TetR) in the presence of doxycycline (Dox) was robust. Expression of shRNAs from engineered human U6 (hU6) promoters containing a single tetracycline operator (TO) sequence between the proximal sequence element (PSE) and the TATA box, or an improved second-generation Tet-responsive promoter element (TRE) placed upstream of the promoter was tight and reversible as judged using quantitative protein measurements. We also established and tested a novel hU6 promoter system in which the distal sequence element (DSE) of the hU6 promoter was replaced with a second-generation TRE. In this system, positive regulation of shRNA production is mediated by novel Tet-dependent transactivators bearing transactivation domains derived from the human Sp1 transcription factor.

**Conclusion:**

Our modified lentiviral vector system resulted in tight and reversible knockdown of target gene expression in unsorted cell populations. Tightly regulated target gene knockdown was observed with vectors containing either a single TO sequence or a second-generation TRE using carefully controlled transduction conditions. We expect these vectors to ultimately find applications for tight and reversible RNAi in mammalian cells in vivo.

## Background

During the past several years, RNAi-based approaches involving small interfering RNAs (siRNAs) and shRNAs have emerged as powerful tools to study gene function in mammalian cells. These RNAs can be expressed intracellularly by cloning shRNA-encoding templates into Pol II or Pol III transcription units [1]. Pol III-based strategies are ideally suited to express shRNAs due to the natural role of Pol III to synthesize small RNAs with precisely defined ends at very high levels, reaching up to 4 × 10^5 ^transcript copies per cell [2].

For many applications, including transgenic approaches involving genes whose inactivation is embryonically lethal, it would be desirable to express shRNAs conditionally. Two general strategies have emerged allowing controllable production of shRNAs in mammalian cells. The first strategy is based on modified Pol II or Pol III promoters, controlled by drug-responsive repressors or transactivators such as Tet-dependent repressors or transactivators [3-17], ecdyson-dependent transactivators [18] or the HIV-1 Tat protein [19]. In an alternative strategy, the Cre-lox recombination system was exploited to turn shRNA synthesis on or off [20-22]. In contrast to the repressor/transactivator-dependent systems, the effects of the Cre-lox system lead to irreversible changes in the vector genome.

Regulatable expression cassettes encoding shRNAs have been delivered into mammalian cells by transient or stable transfection or by using viral vectors such as lentiviral vectors. Alternatively, vectors based on oncogenic retroviruses or adenovirus-based vectors are being used [1,23]. It is evident from the recent literature that the currently available expression systems for regulated shRNA production differ widely in terms of gene silencing efficiency and control thereof. For example, to attain significant knockdown of target gene expression using adenoviral vectors encoding shRNAs corresponding to the c-myc coding region, multiplicities of infection (MOI) up to 3000 were used [10,11]. It is not clear whether the need for such high MOIs was caused by poor transduction efficiencies or ineffective shRNAs, or both.

Tet-regulatable lentiviral vectors encoding shRNAs were first described by Wiznerowicz and Trono [6] and by Miyagishi et al. [9]. In the report by Wiznerowicz and Trono, MOIs of at least 10 were used to attain efficient target gene knockdown. In this case, MOIs were calculated based on titers obtained by flow cytometric analysis (fluorescence activated cell sorting, FACS) of vector-encoded EGFP expression in HeLa cells. Given that measurements based on FACS lead to an underestimation of vector titers, the number of shRNA-encoding vector genomes per target cell may have been up to 20-fold higher [24]. Miyagishi et al. [9] observed robust knockdown of target gene expression using lentiviral vectors at lower MOIs. However, this was only observed following isolation of cell clones. However, selection of cell clones is not always possible particularly in the context of primary cells.

Recently, lentiviral vector systems bearing Tet-reponsive Pol II promoters and shRNA-sequences embedded in a microRNA context were described [14,17]. In these studies, transduced cells expressing high shRNA levels were first enriched by FACS prior to determining the robustness of shRNA-mediated knockdown of target gene expression in such cells. Thus, the usefulness of these systems and their background expression levels in unsorted cell populations remain to be determined.

In this study, we present quantitative data demonstrating that, when the hU6 promoter is engineered to contain a single TO sequence between the PSE and the TATA box, or an additional second-generation TRE upstream of the promoter, knockdown of target gene expression can be tightly and reversibly repressed in the presence of a modified TetR bearing a KRAB silencing domain. We also describe the performance of a novel U6 promoter system in which the DSE of the hU6 promoter was replaced with a second-generation TRE. In this system, positive regulation of shRNA production is mediated by a novel Tet-dependent transactivator, rtTASp1(AB), bearing the transactivator domains A and B of the human Sp1 transcription factor [25].

## Results

### Design of improved lentiviral vectors for conditional, Dox-dependent expression of shRNAs

**Figure 1:**
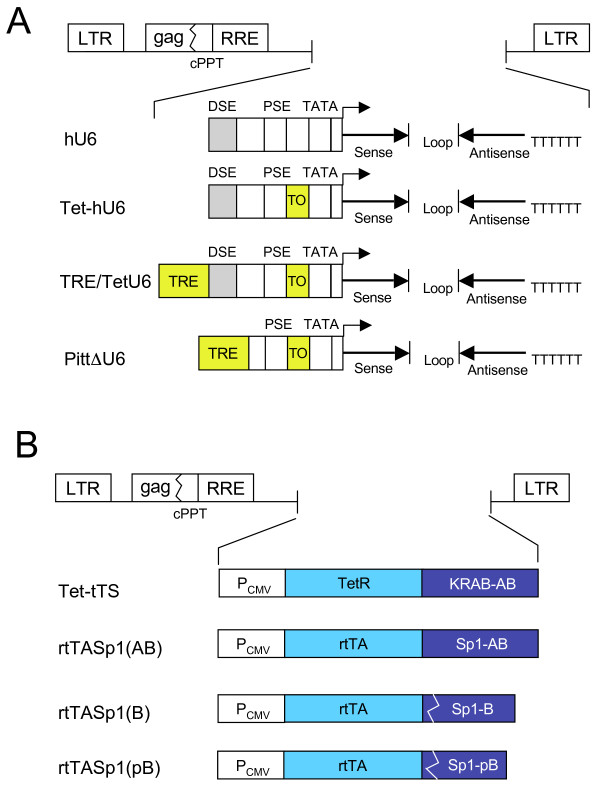
**Schematic representation of lentiviral vectors containing modified U6 promoter sequences or Tet-dependent repressor and transactivator sequences**. (A) Promoters. DSE, distal sequence element; PSE, proximal sequence element; TO, tetracycline operator; TATA, TATA box; hU6, human U6 promoter; Tet-hU6, human U6 promoter with an internal TO; TRE/TetU6, a second–generation TRE, lacking a TATA box and consisting of eight repositioned TO sequences placed upstream of the Tet-hU6 promoter; PittΔU6, hU6 promoter with DSE element deleted and replaced using a second-generation TRE lacking a TATA box. (B) Repressors and transactivators. P_CMV_, human cytomegalovirus immediate early promoter; TetR, tetracycline repressor; KRABAB, KRAB-AB domain of the Kid-1 protein; rtTA, amino acids 1–207 derived from the Tet-controlled rtTA2^S^-M2 reverse transactivator; Sp1-AB, Sp1-B and Sp1-pB, Sp1-derived transactivation domains.

Our goal was to design improved binary Tet-regulatable lentiviral vector systems bearing Tet-responsive Pol III promoters allowing tightly controlled and reversible RNAi-mediated knockdown of target gene expression. To accomplish this, lentiviral vectors were developed harboring Tet-regulatable hU6 promoters controlled either using a chimeric TetR or Tet-dependent transactivators (Figure 1). Several TetR-responsive promoter constructs were designed. In the Tet-hU6 construct, a 22-bp TO sequence was placed between the PSE and the TATA box of the hU6 promoter (Figure 1A). In the TRE/TetU6 construct, a second–generation TRE, lacking a TATA box and consisting of eight repositioned TO sequences [26,27] was placed upstream of the DSE (Figure 1A). For Dox-controlled production of shRNAs from Tet-hU6 and TRE/TetU6 promoter-bearing vectors, a lentiviral vector expressing a chimeric TetR protein containing a KRAB-AB silencer domain [28], referred to as Tet-tTS was developed (Figure 1B).

In a second strategy, Tet-regulatable hybrid hU6 promoters that responded to Tet-dependent chimeric transactivators were designed. In one such promoter construct, referred to as PittΔU6, the hU6 DSE element that supports Pol III transcription [29], was replaced using a second-generation TRE lacking a TATA box (Figure 1A) to make it responsive to Sp1-containing transactivators [30]. For Dox-controlled production of shRNAs from the hybrid PittΔU6 promoter, novel Tet-controlled reverse transactivators bearing glutamine-rich transactivation domains derived from the human Sp1 transcription factor were constructed (Figure 1B). Hybrid rtTASp1 transcription factors bearing the Sp1 AB domain, the B domain or a truncated B domain [31] fused to the DNA binding domain of a reverse Tet-controlled transactivator (rtTA2^s^-M2) [32] were developed. These transactivators are referred to as rtTASp1(AB), rtTASp1(B) and rtTASp1(pB), respectively (Figure 1B).

### Knockdown of target gene expression in cells co-transduced with lentiviral vectors containing the of Tet-hU6 promoter and the Tet-tTS repressor

**Figure 2:**
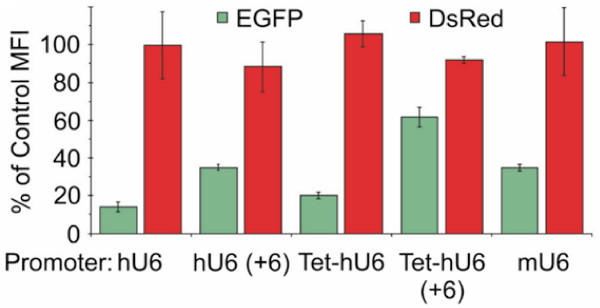
**Test of U6 promoters in HeLa G/R cells**. Changes in relative EGFP expression levels in HeLa G/R cells determined by flow cytometry using lentiviral vectors encoding an shRNA corresponding to EGFP controlled by various U6 promoters. For transduction, cells were exposed to 50 MOI (HOS cell units determined by real-time PCR). Expression levels are represented by MFI values of transduced cells compared to mock transduced cells, which are referred to as percentage of control MFI. Results shown were taken 7 days posttransduction and represent the mean ± SD of at least three experiments. hU6, human U6 promoter; Tet-hU6, human U6 promoter with an internal TO; mU6, mouse U6 promoter.

The ability of lentiviral vectors bearing the modified Tet-hU6 promoter and of vectors bearing the unmodified hU6 promoter to knock down EGFP target gene expression in HeLa G/R cells co-expressing EGFP and DsRed was evaluated first. All virus stocks were adjusted based on titers determined by real-time PCR. Knockdown of EGFP expression was analyzed by FACS. It was found that knockdown of EGFP expression was efficient with the unmodified hU6 promoter leading to a reduction in the mean fluorescence intensity (MFI) of the EGFP-expressing cell population below 15% of that seen in mock-infected HeLa G/R cells (Figure 2). The Tet-hU6 and mU6 promoters were found to be slightly less efficient at the same MOIs, with the MFI levels reduced by some 80% and 70%, respectively relative to those seen in untransduced HeLa G/R cells (Figure 2). When the shRNAs produced from these promoters was modified to include six additional nucleotides at the 5' end, referred to as hU6 (+6) and Tet-hU6 (+6) respectively, knockdown of EGFP expression was reduced compared to that seen with the unmodified constructs (Figure 2). In all cases, DsRed expression levels were not impaired indicating that the knockdown was specific.

**Figure 3:**
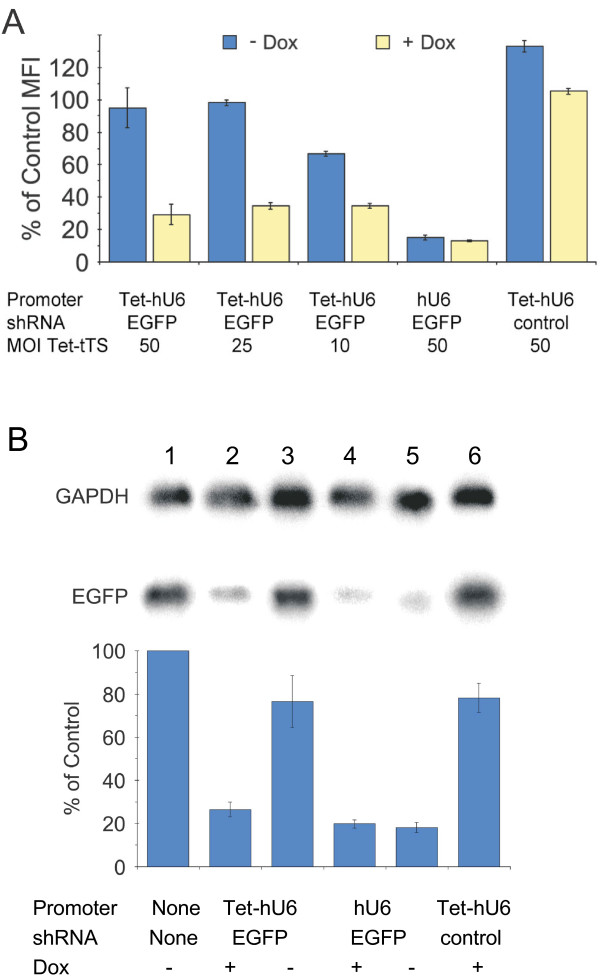
**Regulated silencing of EGFP expression in HeLa G/Rcells**. (A) Control of shRNA expression, as determined by flow cytometry, based on the presence (yellow) or absence (blue) of Dox. HeLa G/R cells were co-transduced with a lentiviral-based silencing construct at an MOI of 25 and lentiviral vectors encoding the Tet-tTS repressor at MOIs varying from 10 to 50 as indicated. An shRNA corresponding to CXCR4 was used as a control. Results shown were taken from cells harvested 7 days posttransduction. (B) Northern blot analysis of 10 μg of total RNA isolated from HeLa G/R cells 7 days posttransduction. Sample 1: Mock control. Samples 2–6 were transduced with 25 MOIs of the lentiviral silencing constructs and 25 MOIs of the Tet-tTS repressor lentivirus. An shRNA corresponding to CXCR4 was used as a control (sample 6). RNA levels seen in the Northern blot were quantified by imaging of phosphor excitation. Results of quantification are shown in the graph. All levels represent the percentage of EGFP compared to the mock control (sample 1) and are based on the EGFP:GAPDH RNA ratios.

Controlled knockdown of EGFP expression was investigated next. To do this, HeLa G/R cells were co-transduced with a constant amount of the shRNA-producing lentiviral vector and variable amounts of the Tet-tTS lentiviral vector. Half of these samples were cultured as normal; the other half were cultured in the presence of Dox. Cells were harvested 7 days after co-transduction, and EGFP expression was analyzed by flow cytometry. EGFP expression in co-transduced cells was then compared to untransduced cells (Figure 3A). It was found that EGFP expression was tightly controlled in cells transduced with the Tet-hU6 vector at Tet-tTS MOIs of 25 and 50 by the inclusion or exclusion of Dox in the culture medium. However, transduction of the Tet-tTS vector at an MOI of 10 did not lead to tight promoter shutoff in the absence of Dox. As expected, a CXCR4-specific control shRNA did not cause any decrease in EGFP levels with or without Dox. These findings were then verified by Northern blot analysis of RNA extracted from HeLa G/R cells co-transduced with Tet-tTS and shRNA-expressing lentiviral vectors at MOIs of 25 each (Figure 3B). Quantitation of the blot revealed that in the presence of Dox, EGFP mRNA levels in cells co-transduced with Tet-hU6 and Tet-tTS decreased by as much as 74% compared to untransduced cells.

To extend our studies, we next attempted to silence expression of the human chemokine receptor CXCR4 gene in a controlled manner. To do this, shRNA-encoding sequences containing 27-bp overlaps with the target sequence [33] were cloned downstream of the Tet-hU6 and TRE/TetU6 promoters present in lentiviral vectors. The ability of such lentiviral vectors encoding shRNAs corresponding to CXCR4 to silence CXCR4 expression in a regulatable manner was assessed by determining CXCR4 protein levels.

**Figure 4:**
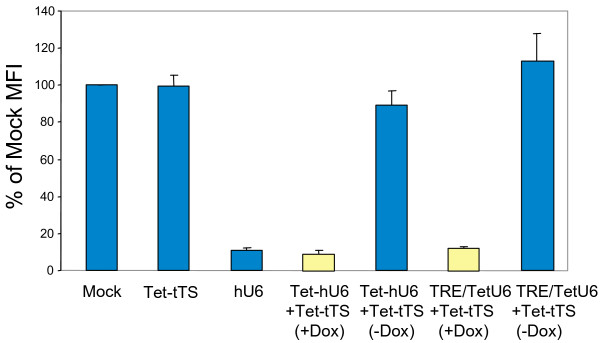
**Regulated knockdown of CXCR4 chemokine receptor expression in HOS-CD4-Fusin cells**. Knockdown of cell surface CXCR4 expression was determined by flow cytometry following transduction of HOS-CD4-Fusin cells with lentiviral vectors expressing Tet-tTS, controlled by the CMV promoter and/or a CXCR4 shRNA, controlled by hU6 promoters. Cells were incubated in the presence or absence of Dox for 6 days. CXCR4 receptor levels were determined by flow cytometry after incubation of the cells with PE-labeled anti-human CXCR4 antibody. The results shown represent the mean ± SD of 3 experiments. They are displayed as the percentage of the MFI of mock-transduced cells.

To do this, HOS-CD4-Fusin cells that overexpress CXCR4 [34] were co-transduced with lentiviral vectors bearing hU6, Tet-hU6 or TRE/TetU6 promoters to drive expression of shRNAs corresponding to CXCR4 mRNA and with a Tet-tTS-expressing lentiviral vector at MOIs of 25 each. Cells were cultured with or without Dox. After 6 days, a flow cytometric analysis was carried out to determine the CXCR4 levels in transduced cells compared to mock-transduced HOS-CD4-Fusin cells (Figure 4). It was found that knockdown of CXCR4 expression was very efficient. There was a 89% ± 1.2%, 91% ± 2.3% and 88% ± 0.9% decrease in the MFI of the cells following transduction with lentiviral vectors bearing hU6, Tet-hU6 or TRE/TetU6 promoters, respectively. In the absence of Dox, CXCR4 receptor levels were unaltered indicating that repression was tight. As expected, expression of the Tet-tTS repressor alone did not affect CXCR4 receptor levels (Figure 4). In agreement with the FACS data, there was a drop in CXCR4 mRNA levels in the presence of Dox but not in the absence as determined by quantitative RT real-time PCR (data not shown).

**Figure 5:**
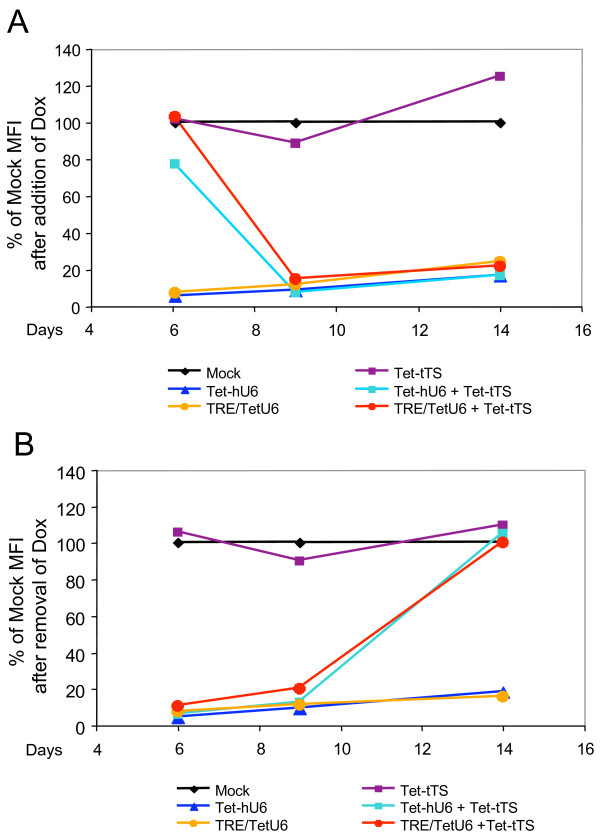
**Time course of CXCR4 knockdown**. (A) HOS-CD4-Fusin cells were co-transduced with lentiviral vectors as described in Figure 4. Transduced cells were incubated in the absence of Dox. On day 6, Dox was added to the culture medium, and cells were analyzed for CXCR4 receptor levels by flow cytometry at various times. (B) HOS-CD4-Fusin cells were co-transduced with lentiviral vectors as described in Figure 4. Cells were incubated in the presence of Dox for 6 days. Dox in the culture medium was then removed and cells were analyzed for CXCR4 receptor levels by flow cytometry at various times.

To show that knockdown of CXCR4 expression in HOS-CD4-Fusin cells is reversible and to establish the kinetics of knockdown, HOS-CD4-Fusin cells were transduced at an MOI of 25 for both the Tet-tTS and shRNA-expressing lentiviral vectors. The transduced cells were then expanded in either the absence (Figure 5A) or the presence (Figure 5B) of Dox for 6 days, at which time Dox was added to the cells previously grown in the absence of it and removed from those previously grown in its presence. The cells were analyzed for CXCR4 expression by flow cytometry. This analysis revealed that, after 6 days of growth in the absence of Dox, CXCR4 expression decreased to almost 11 background levels within 3 days of the addition of Dox (91% ± 0.2% for the Tet-hU6 promoter and 85% ± 1.0% for the TRE/TetU6 promoter) (Figure 5A). However, after removal of Dox from the medium of cells initially cultured in its presence, it took significantly longer for CXCR4 expression to reach control levels (105% ± 8.0% for the Tet-hU6 promoter and 100% ± 8.0% for the TRE/TetU6 promoter) (Figure 5B). This finding suggests that intracellular Dox persisted for some time after removal of Dox from the culture medium.

### Positive control of Pol III promoter activity using novel Tet-dependent transactivators

**Figure 6:**
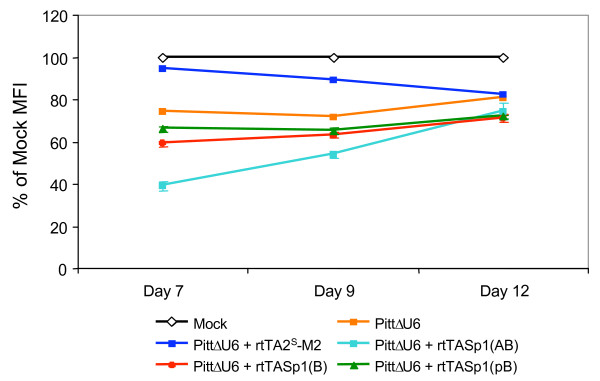
**Positive regulation of Pol III promoter activity using Dox-dependent transactivators bearing Sp1 transactivation domains**. HOS-CD4-Fusin cells were co-transduced with a lentiviral vector expressing an shRNA corresponding to CXCR4 from the PittΔU6 promoter and lentiviral vectors encoding the rtTASp1(AB), rtTASp1(B), or rtTASp1(pB) transactivator, or the rtTA2^s^-M2 transactivator. Transduced cells were expanded in the presence of Dox for 7 days. Dox was then removed and cells were analyzed by flow cytometry at the times indicated. The results shown are representative of 3 similar experiments performed.

We next wanted to investigate a different approach allowing conditional expression of shRNAs from Tet-responsive Pol III promoters using novel Tet-dependent transactivators. To do this, HOS-CD4-Fusin cells were co-transduced with an shRNA-encoding lentiviral vector containing the PittΔU6 promoter (see Figure 1A) and various rtTASp1 transactivator-encoding vectors (see Figure 1B) at an MOI of 25 for each vector. The transduced cells were then incubated in the presence of Dox for 7 days and then analyzed by flow cytometry. The results presented in Figure 6 show that knockdown of CXCR4 expression in cells transduced with lentiviral vectors containing the PittΔU6 promoter-CXCR4 shRNA expression cassette along with lentiviral vectors encoding rtTASp1 transactivators was most efficient with the rtTASp1(AB) transactivator. In this case, CXCR4 expression decreased by 61% ± 2.4% (Figure 6) while shRNAs expressed from a lentiviral vector bearing an unmodified hU6 promoter led to a 82% drop at the same MOI (data not shown). In contrast, when other rtTASp1 transactivator-encoding vectors were used, silencing of the target gene was less pronounced. CXCR4 levels dropped by 41% ± 1.4% and 34% ± 1.4% for the rtTASp1(B) and rtTASp1(pB)-encoding vectors, respectively. Silencing of CXCR4 expression resulting from CXCR4 shRNAs was reversible. Five days after removal of Dox, CXCR4 levels reached those observed with cells bearing PittΔU6 promoter-CXCR4 shRNA cassettes alone (Figure 6). As expected, expression of the rtTASp1(AB) transactivator alone did not change CXCR4 levels. However, in HOS-CD4-Fusin cells singly transduced with a lentiviral vector bearing the PittΔU6 promoter-CXCR4 shRNA expression cassette, there was a 26% drop in CXCR4 levels as judged by flow cytometry indicating that the PittΔU6 promoter is leaky.

As expected, the MFI of cells co-transduced with a lentiviral vector containing the PittΔU6 promoter-CXCR4 shRNA cassette and a lentiviral vector encoding the rtTA2^S^-M2 transactivator [27] in the presence of Dox were unaltered relative to mock-transduced cells (94% of mock MFI). This is consistent with previous findings that transactivators containing Sp1 domains preferentially transactivate Pol III-type promoters while VP16-domain-containing transactivators preferentially interact with mRNA-type promoters [30]. It was interesting to note however, that binding of the rtTA2^S^-M2 transactivator to the PittΔU6 promoter in the presence of Dox reduced the promoter's leakiness (Figure 6). Reduced leakiness of the PittΔU6 promoter-CXCR4 shRNA vector was also observed in cells transduced with the Tet-tTS lentiviral vector (data not shown). Thus, the leakiness observed with the PittΔU6 promoter can be overcome by co-expressing rtTASp1(AB) and Tet-tTS sequences in target cells.

## Discussion

Lentivirus-based vectors provide powerful tools for the introduction of shRNA-encoding sequences into primary cells, ES cells and embryos [35]. However, in general, only a fraction of the transduced cells display detectable RNAi at variable levels [21], indicating that the expression of shRNAs from lentiviral vectors is affected by position effects. This problem can in part be overcome by using high enough MOIs to ensure multiple shRNAencoding cassettes per genome. Among those cassettes, some are expected to escape position-dependent effects, resulting in sustained shRNA expression. Alternatively, isolated cell clones obtained by FACS sorting of transduced cell populations can be used to increase the robustness of shRNA-mediated gene knockdown [9,14,17,36]. Unfortunately, selection of cell clones is not always possible particularly in the context of primary cells. More importantly, selection strategies are in general not feasible in vivo.

To characterize our lentiviral vector system for its capacity to conditionally knock down gene expression, we chose the EGFP transgene as a target for our initial investigation. It was found that silencing of EGFP protein and mRNA production was repressed quantitatively in the absence of Dox provided that a high enough MOI of the two vectors was used. It was also found that in contrast to the findings reported by Lin et al. [12], our single-copy TO configuration allowed tightly regulated EGFP knockdown. The improved performance of our system compared to that reported by Lin et al. may be related to the fact that a TetR bearing the potent KRAB silencing domain was used in our work. In contrast, Lin et al. used an unmodified TetR. To fully validate the two systems, it will ultimately be necessary to compare shRNA levels in transduced cells in the presence and absence of Dox using Northern blot experiments.

As a second target, we chose the CXCR4 chemokine receptor gene. In our Tet-regulatable lentivirus system, robust CXCR4 knockdown was observed in unsorted cell populations at MOIs of 25. The excellent performance of our system may be due in part to the design of the shRNAs. It was recently reported that synthetic dsRNAs and shRNAs longer than 19–21 bp were able to more efficiently enter the RNAi pathway and to knock down the target gene without causing nonspecific effects [33,38]. Based on these findings, we used CXCR4-specific shRNAs, 27 nt in length.

It is evident from Figures 2 and 3 that insertion of a single TO sequence upstream of the TATA box had only a minor effect on the performance of the Tet-hU6 promoter. Also, the experiments presented in Figures 4 and 5 show that RNAi mediated by the Tet-hU6 and TRE/TetU6-containing vectors was tight in the absence of Dox. This indicates that the two systems performed equally well as far as HOS-CD4-Fusin cells are concerned.

The position of the TRE element appears to influence background expression. Recent evidence presented by Zhou et al. [39] has shown that the distance between the TRE element and the promoter sequence is important as far as Pol II promoters are concerned. TRE elements placed in close proximity to a Pol II promoter sequence displayed lower background expression compared to TRE elements placed farther away within the viral LTRs. It remains to be determined whether the same is true for Pol III promoters.

With a view toward designing Pol III promoters that respond to Tet-dependent transactivators, we developed an alternative Tet-dependent lentiviral vector system. In this system, novel Dox-dependent transactivators consisting of Sp1 transcription factor transactivation domains fused to a truncated version of the rtTA2^S^-M2 protein lacking the VP16 transactivation domain were expressed from lentiviral vectors. Expression of shRNA is achieved from a hybrid U6 promoter lacking the DSE. Using this system, Dox-dependent knockdown of CXCR4 expression was clearly evident as shown in Figure 6. However, there was background expression in the absence of the transactivator and/or Dox. Interestingly, cells co-transduced with a lentiviral vector bearing the PittΔU6/shRNA-encoding sequences and a second lentiviral vector encoding the rtTA2^S^-M2 transactivator in the presence of Dox displayed lower background expression possibly due to the fact that the rtTA2^S^-M2 transactivator blocked Pol III activity. Reduced background expression was also observed in cells bearing the Tet-tTS repressor (data not shown). It is conceivable that cells displaying lower background expression could also be isolated by FACS sorting or drug selection. Such a strategy was used by Gupta et al. [18] to isolate ecdyson-inducible cell clones expressing shRNA from hybrid RNA Pol III promoters in response to a GAL4-Oct2^Q^(Q→A) transactivator. While this manuscript was in preparation, Amar et al. [40] described a regulatable lentiviral vector system containing a hybrid transactivator consisting of the Oct2^Q^(Q→A) transactivation domain fused to the rtTA2^S^-M2 DNA binding domain and a U6 core promoter preceded by 7 TO sequence. Similar to our system, this system was found to be leaky in the absence of Dox. However, isolated clones displayed low background expression and robust inducibility.

For tight and reversible RNAi in vivo, it would be desirable to have combined vectors available harboring Tet-dependent repressors or transactivators as well as shRNA-encoding sequences. Attempts to generate such vectors have been reported by others [36,40]. However, these vectors appear to be leaky.

## Conclusion

We provide quantitative data suggesting that robust and tightly regulated knockdown of gene expression can be obtained using lentiviral vectors bearing either a single TO sequence or a second-generation TRE in transduced cell populations without the need for sorting cell clones.

## Methods

### Cell lines

Cell lines used included human embryonic kidney 293 T cells (ATCC, CRL-11268) [41], human osteosarcoma (HOS) cells (ATCC, CRL-1543), HOS-CD4-Fusin cells (provided by Dr. Nathaniel Landau through the NIH AIDS Research and Reference Reagent Program, Germantown, MD) [34] and HeLa cells (ATCC, CCL-2). HeLa cells co-expressing EGFP and DsRed (referred to as HeLa G/R cells) were generated by co-transduction of HeLa cells with NL-CMV/EGFP and NL-CMV/DsRed lentiviral vectors [42]. Cells were maintained in Dulbecco's modified Eagle's medium (DMEM) (Gibco, Grand Island, NY) and supplemented with 10% heat-inactivated fetal bovine serum (FBS) (HyClone, Logan, UT).

### Construction of lentiviral vectors

The hU6 promoter was amplified from HeLa cell DNA by polymerase chain reaction (PCR) as described [43] and cloned into the EcoRI and XbaI sites of pUC19 to yield pUC-U6. To generate a wild-type hU6 promoter and a hU6 promoter harboring the TO sequence with an ApaI site for cloning of shRNA, the following mutually priming oligonucleotides were used: WT sense (5'-CATATGCTTACCGTAACTTGAAAGTATTTCGATTTCTTGGCTTTATATATCTTGTGGAAAGGACGAA ACACCG-3'), Tet sense (5'-CATATGCTTACCGTAACTTGAAAGTACTCTATCATTGATAGAGTTATATATCT TGTGGAAAGGACGAAACACC-3'), and antisense (5'-TCTAGACTCGAGAATAGTGTTGTGTGCCTAGGATATGTGCTGCCGAAGCGGG CCCGGTGTTTCGTCCTTTCCACAA-3'). Annealed oligonucleotides were filled in using the Sequenase v. 2.0 DNA polymerase (USB, Cleveland, OH) and subsequently were cloned into the NdeI and XhoI sites of pUC-U6, resulting in pUC-hU6 (+6) and pUC-Tet-hU6 (+6), respectively. To move the ApaI site immediately upstream of the start of transcription (position +1), a PCR was performed using a sense primer (5'- CGCCAGGGTTTCCCAGTCACGAC-3') and a mismatched antisense primer (5'- TCTAGACTCGAGGGGCCCTCGTCCTTTCCACAAGATATAT-3'). The resulting fragments were cloned into pUC19 to yield pUC-Tet-hU6 and pUC-hU6. To construct a derivative of the Tet-hU6 promoter harboring an upstream TRE, a 300-bp PCR fragment containing a TRE/Pitt promoter sequence [26,27] lacking a TATA box sequence was PCR amplified using pNL-TRE/Pitt-EGFPΔU3 DNA [27] as a template. The following primers were used: sense primer (5'-CCGATGATATCAAGTGCCACCTGACGTCTCCCTA-3') and antisense primer (5'- TCGTGGCTAGCCTCTATCACTGATAGGGAGCTCG-3'). The resulting PCR fragment was first cloned into the pCR4-TOPO plasmid (Invitrogen, Carslbad, CA), resulting in the pCR-TRE plasmid. The insert fragment was cut out using EcoRI and subsequently ligated into the unique EcoRI site present in pUC-Tet-hU6, generating the pUC-TRE/Tet-U6 plasmids. A truncated version of the U6 promoter containing a TRE sequence replacing the DSE element was constructed as follows: A 695-bp PCR fragment was prepared using a sense primer (5'-GATCCGCTAGCGCTGTTAGAGAGATAATTAG-3') and an antisense primer (5'- GTCAGCACTAGTGGTACCCGGAGCCTATGGAAAAACG-3') and plasmid pUC- hU6 as a template. The resulting PCR fragment containing a U6 promoter lacking the DSE sequence was subcloned into the pCR-TRE plasmid using the NheI and SpeI sites, resulting in pPittΔU6.

To introduce an shRNA-encoding cassette downstream of the U6 promoter sequence, mutually priming oligonucleotides corresponding to the EGFP sequence were cloned into the ApaI and XhoI sites of the various plasmids bearing hU6 or Tet-hU6 promoter sequences or the murine U6 (mU6) promoter which was derived from the pSilencer1.0 plasmid (Ambion, Austin, TX). Sequences corresponding to CXCR4 were cloned downstream of the hU6, Tet-hU6, TRE/TetU6 and PittΔU6 promoters. The sequences of the mutually priming oligonucleotides used were as follows: EGFP sense (5'- GGGCCCGCAAGCTGACCCTGAAGTTCcttcctgtcaGAACT-3'), EGFP antisense (5'- CTCGAGAAAAAAGCAAGCTGACCCTGAAGTTCtgacaggaagGAACT-3'), CXCR4 sense (5'-CGGATCAGTATATACACTTCAGATAACTaagttctctAGTTATCTGAAGTGTATATACTGATCCTTTTTC-3'), and CXCR4 antisense (5'-TCGAGAAAAAGGATCAGTATATACACTTCAGATAACTagagaacttAGTTATCTGA AGTGTATATACTGATCCGGGCC-3'). The lowercase letters in these sequences refer to the loop sequence in the resulting hairpin RNA, whereas the capital letters represent the sequences corresponding to the target mRNA. The sequence targeting EGFP mRNA is 19 nucleotides (nt) long and the sequence targeting CXCR4 is 27 nt long. The promoters and shRNA-encoding cassettes of the resulting plasmids were sequenced to verify fidelity.

To create lentiviral vectors bearing U6 promoter-shRNA cassettes, sequences were excised from the pUC backbone using EcoRI, blunted using T4 DNA polymerase (New England Biolabs, Beverly, MA), and cleaved using XhoI. This fragment was cloned into the HpaI and XhoI sites of the pNL-EGFP/CMV lentiviral vector [44,45]. To create lentiviral vectors bearing the TRE/TetU6 promoter-shRNA cassette, sequences were excised from the pUC-TRE/Tet-U6 backbone using EcoRV and PstI, and blunted using T4 DNA polymerase. This fragment was then cloned into the HpaI and XhoI (blunted) sites of the pNL-EGFP/CMV lentiviral vector. In order to obtain lentiviral vectors bearing the PittΔU6 promoter-shRNA cassette, the corresponding sequences were excised from pPittΔU6 using EcoRV and KpnI and ligated into the HincII/KpnI site of pNL-EGFP/CMV.

Plasmid pNL-rtTA2^S^-M2 containing the rtTA2^s^-M2 transactivator sequence controlled by the constitutive human CMV-IE promoter was described previously [27]. A lentiviral plasmid encoding the chimeric Tet-tTS TetR [28] was generated as follows: pTet-tTS (Clontech, Palo Alto, CA) was cut using ClaI, blunted, and then cut using XbaI. The resulting 850-bp fragment was ligated into the XbaI and BamHI (blunted) sites of the pNL-rtTA2^S^-M2 lentiviral vector [27] to yield pNL-Tet-tTS. Lentiviral vectors encoding three variants of the rtTASp1 transactivator were generated as follows: A 1250-bp fragment encoding the glutamine-rich A and B domains (amino acids 80–485) of the Sp1 transcription factor [25,31,46] was PCR amplified using a sense primer (5'- attgtcgatatcggccggaggaggatcccagggcccgagtcagtca-3') and an antisense primer (5'- agataacccgggtgctaaggtgattgtttgggcttgt-3'). A 690-bp fragment encoding the Sp1 B domain (amino acids 263–485) was PCR amplified using a sense primer (5'attgtcgatatcggccggaggaggatccatcaccttgctacctgtcaa-3') and the same antisense primer as before. Finally, a 290-bp fragment encoding the C-terminal part (amino acids 398–485) of the Sp1 domain B was PCR amplified using a sense primer (5'attgtcgatatcggccggaggaggatctcttatccagcctcagctagt-3') and the same antisense primer as before. In all cases, template DNAs for PCR were prepared by reverse transcription of human total RNA (Human Control RNA, Applied Biosystems). The PCR fragments were cloned into the pCR4-TOPO plasmid (Invitrogen). The resulting plasmids were cut with XmaI and EagI and sequences encoding Sp1 domains were used to replace a 116-bp XmaI/EagI fragment present in pNL-rtTA2^s^-M2 resulting in plasmids pNL-rtTASp1(AB), pNL-rtTASp1(B), and pNL-rtTASp1(pB), respectively.

### Production and titration of lentiviral vectors

Lentiviral vector preparations were generated by calcium phosphate-mediated transfection of 293 T cells with modifications as described [47,48]. Vector stocks were titrated using HOS cells. Cells in six-well plates were transduced with viral vector stocks for 16 h in the presence of 8 μg/ml polybrene. After 16 h, virus-containing medium was removed and replaced with 2 ml of fresh medium. DNase I (Sigma, St. Louis, MO) was added 48 h later directly to the cell culture medium at a final concentration of 2.5 U/ml. After incubation at 37°C for 30 min, the cells were trypsinized, and the genomic DNA as harvested using a DNeasy kit (Qiagen, Valencia, CA). Real-time PCR was then used to quantify the proviral DNA. A primer-probe set corresponding to the viral gag region was used as previously described [27,49]. In parallel, a primer-probe set specific for RNase P (Applied Biosystems) was used to quantify the genomic DNA. Standard curves were generated for RNase P using serial dilutions of total human DNA (Applied Biosystems). The standard curves for gag were generated by serially diluting the pNL-EGFP/CMV plasmid DNA quantified using the Fluorescent DNA Quantification Kit (Bio-Rad Laboratories, Hercules, CA). In all experiments, 1 × 10^5 ^cells were transduced for 16 h in the presence of 8 μg/ml polybrene. The MOIs used are indicated in the Results section. When applicable, Dox was added to the cells at the times indicated at a final concentration of 1 μg/ml.

### Analysis of EGFP and CXCR4 expression by flow cytometry

Transduced cells expressing EGFP were trypsinized and collected and washed twice with PBS containing 2% FBS and analyzed by FACS [50]. For antibody staining, trypsinized cells were resuspended in PBS/2% FBS containing phycoerythrin-labeled anti-human CXCR4 monoclonal antibody (Clone 12G5, R&D Systems, Inc., Minneapolis) and incubated for 1 h at room temperature. Cells were washed twice with PBS/2% FBS and analyzed by FACS.

### Northern blotting of cellular RNA

Total RNA was isolated from HeLa G/R cells using Trizol reagent (Invitrogen, Carlsbad, CA). RNA (10 μg) was blotted and probed for EGFP and glyceraldehyde-3-phosphate dehydrogenase (GAPDH) sequences. Northern blot analysis was done as described before [27].

### Quantitative reverse-transcriptase real-time PCR

Total RNA was isolated from HOS-CD4-Fusin cells using Trizol reagent (Invitrogen). For one-step reverse-transcriptase real-time PCR (RT real-time PCR), 50 ng of total RNA per reaction was used. Reactions were performed as described in the Applied Biosystems protocol for one-step RT real-time PCR. To quantify CXCR4 mRNA levels, a commercially available primer-probe set (FAM labeled) was used (TaqMan Gene Expession Assays, HS00237052m1, Applied Biosystems). To quantify the levels of the reference GAPDH mRNA, a target-specific primer-probe set (VIC labeled) was used (Human Endogenous Controls, catalog number: 4310884E, Applied Biosystems). Reactions were performed using the Mx3000P™ Real-Time PCR System (Stratagene).

## Competing interests

The author(s) declare that they have no competing interests.

## Authors' contributions

KP, TD, XYZ, RK and AB performed the experiments. JR designed and coordinated the study, and wrote major parts of the manuscript. KP and TD participated in the design of the study.
